# Insights into Membrane Damage by *α*-Helical and *β*-Sheet Peptides

**DOI:** 10.3390/biom15070973

**Published:** 2025-07-07

**Authors:** Warin Rangubpit, Hannah E. Distaffen, Bradley L. Nilsson, Cristiano L. Dias

**Affiliations:** 1 Department of Physics, New Jersey Institute of Technology, Newark, NJ 07102-1982, USA; 2 Department of Chemistry, University of Rochester, Rochester, NY 14627-0216, USA; 3 Materials Science Program, University of Rochester, Rochester, NY 14627-0166, USA

**Keywords:** peptides, lipids, amyloids, antimicrobial, self-assembly, pore-like structure, water permeation

## Abstract

Peptide-induced disruption of lipid membranes is central to both amyloid diseases and the activity of antimicrobial peptides. Here, we combine all-atom molecular dynamics simulations with biophysical experiments to investigate how four amphipathic peptides interact with lipid bilayers. All peptides adsorb on the membrane surface. Peptide M01 [Ac-(FKFE)_2_-NH_2_] self-assembles into β-sheet nanofibrils that span both leaflets of the membrane, creating water-permeable channels. The other three peptides adopt α-helical structures at the water–lipid interface. Peptide M02 [Ac-FFKKFFEE-NH_2_], a sequence isomer of M01, does not form β-sheet aggregates and is too short to span the bilayer, resulting in no observable water permeation across the membrane. Peptides M03 and M04 are α-helical isomers long enough to span the bilayer, with a polar face that allows the penetration of water deep inside the membrane. For the M03 peptide [Ac-(FFKKFFEE)_2_-NH_2_], insertion into the bilayer starts with the nonpolar N-terminal amino acids penetrating the hydrophobic core of the bilayer, while electrostatic interactions hold negative residues at the C-terminus on the membrane surface. The M04 peptide, [Ac-FFKKFFEEFKKFFEEF-NH_2_], is made by relocating a single nonpolar residue from the central region of M03 to the C-terminus. This nonpolar residue becomes unfavorably exposed to the solvent upon insertion of the N-terminal region of the peptide into the membrane. Consequently, higher concentrations of M04 peptides are required to induce water permeation compared to M03. Overall, our comparative analysis reveals how subtle rearrangements of polar and nonpolar residues modulate peptide-induced water permeation. This provides mechanistic insights relevant to amyloid pathology and antimicrobial peptide design.

## 1. Introduction

Disruption of lipid membranes by amphipathic peptides and their aggregates contributes to cytotoxic effects in amyloid diseases such as Alzheimer’s disease by inducing neuronal damage, resulting in cognitive and motor decline [1,2,3,4]. This phenomenon also plays a key role in host defense, as many organisms express amphipathic peptides that target bacterial membranes, while selectively sparing eukaryotic cells [5,6,7]. This selectivity has motivated efforts to develop amphipathic peptides as therapeutic agents for bacterial infections, and to target and destroy cancer cells [8,9,10,11]. These empirical efforts have provided only limited insight into the detailed interactions and pathways that cause membrane disruption. Greater mechanistic understanding will lead to the discovery of principles to guide the design of novel therapeutic agents [12,13,14,15]. Insights into these principles can be obtained via high-throughput analyses of peptide databases, and all-atom simulations combined with validating biophysical/biochemical experiments.

In 2025, nearly 3500 antimicrobial peptides (AMP) have been reported in the literature [16,17]. Most of them exhibit a net positive charge (88%), with only a small fraction being neutral (6%) or negative (6%) [18]. These observations likely reflect the necessity of charge complementarity for positive AMPs to target negatively charged components of bacterial membranes [19]. In contrast, amyloid peptides often only display small and often variable net charges, which facilitates aggregation and self-assembly. Moreover, for amyloids and AMPs, approximately 40–60% of the residues are nonpolar [18]. Hydrophobic amino acids drive the aggregation of amyloid peptides and are critical to account for membrane adsorption [20]. The distribution of these residues along the peptide sequence determines the conformation of peptides at the water–lipid interface. AMPs show a preference for adopting α-helical (16%) compared to β-sheet (2%) structures [16], which is often encoded in sequences containing variations of the (XXYY)_*n*_ template [21,22,23], where X and Y correspond to hydrophobic and polar/charged residues, respectively, and n varies between 2 and 4. In contrast, amyloids self-assemble into β-sheet structures that can be encoded in various sequence patterns, including those where nonpolar and polar/charged residues alternate [24,25,26,27], for example, (XY_1_XY_2_)_*n*_.

The (XXYY)_*n*_ and (XY_1_XY_2_)_*n*_ sequence templates can adopt secondary structures that are amphiphilic, with one of their faces being mostly nonpolar and the other polar. At the water–lipid interface, the former is buried inside the acyl-tail of lipids, whereas the latter is exposed to the solvent and lipid head groups [20]. Some peptides with these sequence patterns tend to damage lipid bilayers [21,22,23,24,28,29,30], which accounts for the increase in the rate of solvent permeation and disrupts ion homeostasis leading to cell death. This damage can manifest via a self-assembly process in which peptides form *pore-like structures*, accumulate in one of the membrane’s leaflets via a *carpet-like mechanism*, or dissolve the lipid content of the membrane in a *detergent-like manner* [31,32,33,34].

Several properties of the amino acid sequence can significantly affect the membrane-damaging nature of AMPs and amyloids. This includes the length of the peptides, which, in some cases, have to be long enough to span the two leaflets of the bilayer [35,36,37]. Increasing the amphiphilicity of peptides enhances secondary structure stability, which can increase the rate of water permeation [15,38,39,40,41]. In the case of α-helices, this includes taking into account that a complete turn contains 3.6 amino acids, and not 4 as assumed in the (XXYY)_*n*_ pattern [39]. Sequence patterns that perfectly segregate polar–nonpolar residues in α-helices are (XXYYXXY)_2_ and (XXYY)_2_(XYYX)_2_. Finally, the hydropathy of the C-terminus has been shown to play an important role in accounting for the rate of water permeation. In particular, amidation of the C-terminus was reported to increase water permeation in several peptides [42,43,44,45] and it is the natural form of many natural AMPs [46]. This effect of C-terminal amidation is not completely understood [42] and could emerge from the stabilization of α-helical conformations due to an additional hydrogen bond or/and from the neutral charge of the amidated C-terminus compared to a peptide with a carboxylic acid at the C-terminus.

In this study, we examine the interactions of four peptides with an anionic lipid membrane to elucidate how water permeation is influenced by the type, length, and amphiphilicity of a peptide’s secondary structure, as well as the hydropathy of its C-terminus. Peptides M01 [Ac-(FKFE)_2_-NH_2_] and M02 [Ac-FFKKFFEE-NH_2_] are isomers with (XY_1_XY_2_)_2_ and (XXYY)_2_ sequence motifs that favor β-sheet and α-helical conformations, respectively. In contrast to M02 peptides, isomers M03 [Ac-(FFKKFFEE)_2_-NH_2_] and M04 [Ac-FFKKFFEEFKKFFEEF-NH_2_] form extended α-helices capable of spanning both bilayer leaflets. Peptides M03 and M04 differ in their C-terminal composition, which is made of only charged residues for M03 but contains a nonpolar residue for M04. Moreover, M04 exhibits a more pronounced segregation of polar and nonpolar residues at the membrane interface, following the (XXYY)_2_(XYYX)_2_ template, while M03 retains the original (XXYY)_4_ pattern. This study was carried out by first using long unbiased all-atom molecular dynamics simulations to predict the propensity of each peptide to induce water permeation and to uncover the underlying molecular mechanisms of this phenomenon. These predictions were subsequently validated by experimental dye leakage assays.

## 2. Materials and Methods

### 2.1. Design of α-Helical Peptides

**Figure 1:**
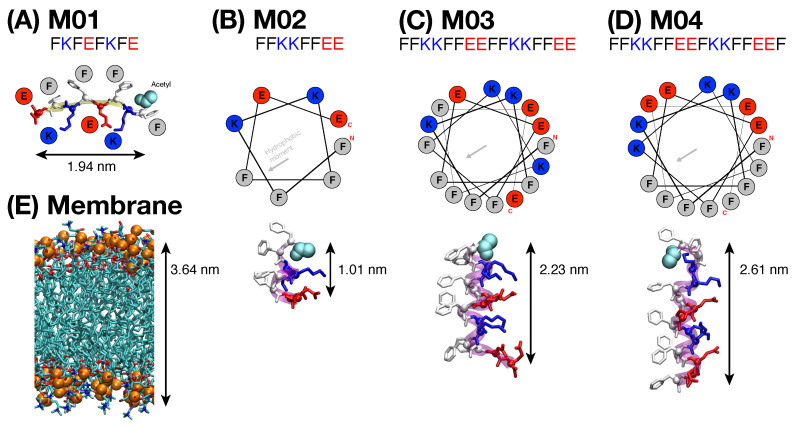
Secondary structures and characteristic lengths of peptides (**A**) M01, (**B**) M02, (**C**) M03, and (**D**) M04. Lysine (K, blue) and glutamic acid (E, red) represent positive and negative charges, respectively. In the helical wheels of M02 and M04, a clear boundary separates non-polar phenylalanine (F, grey) from charged residues, unlike in M03. The end-to-end distances of the peptides are shown. (**E**) Molecular structure of the lipid bilayer with its average thickness of 3.64 nm.

This work was inspired by two 8-residue peptides made from the permutation of 4 phenylalanines (F), 2 lysines (K), and 2 glutamic acids (E). These peptides, labeled M01 (Ac-FKFEFKFE-NH_2_) [20,29] and M02 (Ac-FFKKFFEE-NH_2_) [28], segregate nonpolar and charged side chains to diametrically opposite sides when folded in a β-sheet and α-helix, respectively. Consequently, they exhibit large hydrophobic moments [47] (0.83 and 0.7, respectively) when folded in these secondary structures. Recently, we have shown that both peptides adsorb on the surface of a 7:3 POPC–POPG lipid membrane [20,28]. Moreover, M01 peptides spontaneously self-assemble into large β-sheets that become inserted into the dry core of the lipid bilayer forming a barrel-like structure with water in its interior. In contrast, M02 peptides remain dispersed on the membrane surface, without inserting themselves into it. This difference in behavior may be rationalized by the length of the hydrophobic face of the β-strand and the α-helix, which is 2 nm and 1 nm, respectively—Figure 1A,B. As a result, the hydrophobic face of the β-strand (but not of the α-helix) can span more than one leaflet of the lipid bilayer.

Here, we also design two peptides that have twice the number of amino acids (16 residues) as M02 and can segregate charged and nonpolar residues to opposite directions when folded in a α-helix. Accordingly, the distance spanned by nonpolar residues along the main axis of the α-helix is twice that of the M02 peptides, and similar to that in M01 peptides (∼2 nm)—see Figure 1. The M03 peptide is composed of two consecutive M02 sequences (Ac-(FFKKFFEE)_2_-NH_2_). This sequence pattern does not optically allow a sharp partition of polar and nonpolar residues at the water–lipid interface (Figure 1C), and results in a hydrophobic moment of only 0.53. The M04 peptide (Ac-FFKKFFEEFKKFFEEF-NH_2_) is a correction to this deficiency, whereby one phenylalanine from the middle of the M03 sequence is shifted to the C-terminus. This gives the M04 sequence a hydrophobic moment of 0.87, where charged and nonpolar residues are sharply partitioned to opposite faces of the α-helix.

### 2.2. Simulation Setup

All-atom molecular dynamics simulations were performed using GROMACS 2021 [48,49] with the atomic interactions modeled via the CHARMM36m force field that provides robust parameterization for both peptides and lipid systems [50]. The bilayer contained 200 lipids that were either 1-palmitoyl-2-oleoyl-sn-glycero-3-phosphocholine (POPC) or 1-Palmitoyl-2-oleoyl-sn-glycero-3-phosphoglycerol (POPG). It was made using a 7:3 POPC–POPG lipid ratio, which is commonly employed to study biologically relevant membrane.

The initial box size for the simulations was approximately 12 × 12 × 12 nm^3^, and it contained ∼20,000 TIP3P water molecules. Prior to peptide insertion, the lipid bilayer was equilibrated at 350 K under NVT and NPT conditions for a total of 500 ns to ensure membrane stability.

The leap-frog algorithm was used to integrate the equations of motion, with a time step of 2 femtoseconds. Long-range electrostatic interactions were treated using the Particle Mesh Ewald (PME) technique, with a Fourier grid spacing of 0.12 nm and a short-range cut-off of 1.2 nm [51]. Van der Waals forces were smoothly switched to zero between 1.0 nm and 1.2 nm. A Nośe–Hoover thermostat (τ_*T*_ = 1 ps) was used to maintain the temperature of the membrane, solvent, and peptides at 350 K [52,53]. A semi-isotropic Parrinello–Rahman barostat (τ_*P*_ = 5 ps) was used to maintain the pressure in the system at 1 atm [54,55].

**Figure 2:**
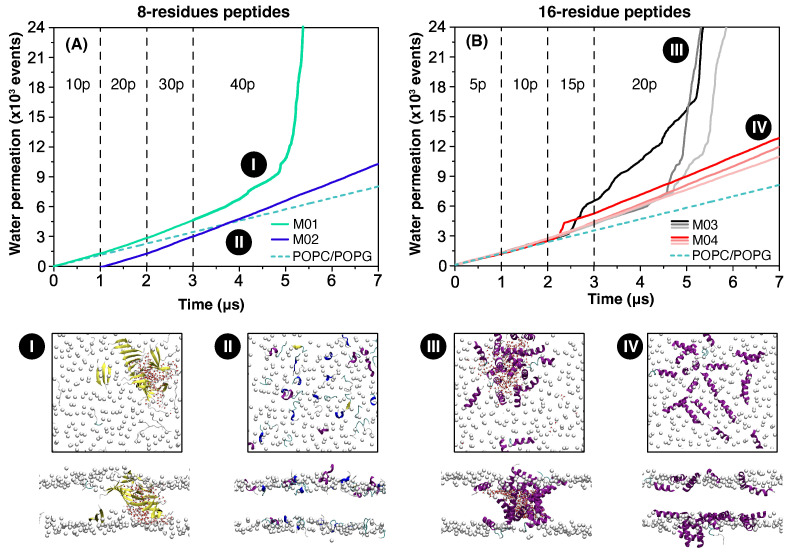
Accumulated water permeation in peptide–membrane simulations. (**A**) M01 [Ac-(FKFE)_2_-NH_2_] in cyan and M02 [Ac-FFKKFFEE-NH_2_] in blue. (**B**) M03 [Ac-(FFKKFFEE)_2_-NH_2_] in black/gray and M04 [Ac-FFKKFFEEFKKFFEEF-NH_2_] in red/pink. Dashed lines indicate permeation in control simulations using a 7:3 POPC–POPG membrane without peptides. Panels I–IV show top and cross-sectional views of the membrane–peptide systems at the end of the simulation. Phosphate atoms are shown as gray spheres; β-strands in yellow, α- and π-helices in purple and blue, and coil regions as gray lines. Water molecules within the bilayer are shown in panels I and III.

Molecular dynamics simulations were performed in four steps of 1 µs, designed to mimic gradual adsorption of peptides onto the membrane [28]. At the beginning of each of these steps, 0.4 amino acids per lipid were randomly added to the solution, which accounted for ten M01 or M02 peptides, and five M03 or M04 peptides. At the end of each 1 µs step, the peptides were found to adsorb onto the membrane, which accounted for 320 amino acids in the last simulation step, i.e., forty M01 or M02 peptides and twenty M03 or M04 peptides. At the end of the fourth step, the simulations were extended by an additional 3 µs to study peptide aggregation and pore formation on the membrane. Three independent simulations were performed for M03 and M04 peptides, showing a consistent membrane permeation effect– Figure 2. An additional 1 µs step was also simulated for the M04 peptides to show its membrane damaging effect at higher concentrations, consistently with the experiments.

The adopted simulation protocol favored a pathway in which peptides first adsorb onto the membrane before self-assembling. This pathway is expected to dominate in experiments performed at low concentration, where the membrane behaves as a sink that gradually adsorbs peptides. This pathway may also be dominant in amyloid diseases, where peptides are initially found in healthy individuals at nano-molar concentrations, i.e., below the critical concentration for fibril formation. It is only over the time-frame of several years that this concentration reaches the micromolar range in the case of the Aβ protein in the brain [56,57,58]. However, alternative pathways of membrane damage are also possible due to the complexity of in vivo conditions [2,59]. Notice that most computational studies start with peptides already embedded onto the membrane [60,61,62], which can lead to biases.

Water permeation was computed using the perm-md-count code that is publicly available on git-hub [63]. It defines a permeation event as the translocation of one water molecule across the entire lipid bilayer along the membrane normal (z-axis), moving from one side of the membrane to the other. Before analysis, the membrane is centered along the z-axis using MDAnalysis, and only the phosphorus atoms of the lipid headgroups within a layer around the membrane midplane are used to define the bilayer region. This setup allowed the robust identification of water molecules that fully crossed the membrane.

### 2.3. Experiments

#### 2.3.1. Peptide Synthesis

Peptides were synthesized using standard Fmoc-protected solid-phase peptide synthesis techniques. Fmoc-protected amino acids (Aapptec) were used in 4x excess and were dissolved in *N,N*-dimethylformamide (DMF) and activated with a great excess (≈17x) of *N,N*-diisopropylethylamine (DIPEA). Amino acids were allowed to activate for 10 min, before addition to deprotected Fmoc-Rink Amide OctaGel Resin (Aapptec, 120–150 mesh, 1% DVB, 0.6 mmol/g, Louisville, KY, USA). Deprotection was performed with 20% *v/v* piperidine/DMF. Manual peptide synthesis was performed in a 75 °C water bath, with the deprotection steps lasting 3 min and the coupling steps lasting 5 min. After the addition of the final amino acid, the Fmoc groups were removed, and the N-termini were acetylated using a solution of 20% *v/v* acetic anhydride in DMF for 3 min in the water bath. After acetylation, the peptide was cleaved from the resin, and side-chain protecting groups were removed using a solution of 95/2.5/2.5% *v/v* trifluoroacetic acid (TFA, Oakwood)/triisopropylsilane (Oakwood)/water for one hour at room temperature. This process was then repeated for another hour after the addition of fresh cleavage cocktail to the resin. The resulting peptide solution was concentrated, and ice-cold diethyl ether was added to precipitate the peptide. The peptide was collected via centrifugation, and the supernatant was decanted. The peptide was dissolved in 60% *v/v* acetonitrile/water with 0.1% TFA, frozen, and lyophilized.

#### 2.3.2. Peptide Purification and Characterization

Purification of the peptides was performed via preparatory scale reverse-phase high-performance liquid chromatography (RP-HPLC) on an Interchim Puriflash 4125 instrument equipped with a Phenomenex Gemini column (10 micron, C18 Axia, 250 × 50 mm, Phenomenex, Torrance, CA, USA). A binary gradient of acetonitrile and water with 0.1% trifluoroacetic acid (TFA) was used as the mobile phase at a flow rate of 100 mL/min (see Table S1 for complete purification conditions) to separate the desired peptides from impurities. The eluent was monitored at a UV absorbance of 215 and 254 nm for fraction collection. Peptide identity and purity were confirmed using matrix-assisted laser desorption ionization-time-of-flight (MALDI-TOF) mass spectrometry (Shimadzu Axia Performance, Shimadzu Corporation, Nakagyo-ku, Kyoto, Japan) and analytical HPLC using a reverse-phase Phenomenex Gemini (5 micron, C18, 110 Å, 250 × 4.6 mm, Phenomenex, Torrance, CA, USA) column on a Shimadzu LC-2010A (Shimadzu Corporation, Nakagyo-ku, Kyoto, Japan). See Figures S6–S8 for MALDI-TOF-MS spectra and Table S2 for tabulated MALDI data. Figures S9–S11 and Table S3 contain analytical HPLC traces of each peptide and the analytical gradient conditions, respectively.

Peptide concentration was determined by dissolving lyophilized peptide powder in 60% *v/v* acetonitrile/water with 0.1% TFA to prevent assembly and analyzing via analytical HPLC for comparison to a concentration curve calibrated by amino acid analysis (UC Davis, Davis, CA, USA). (Representative concentration curves can be found in Figures S12 and S13). Appropriate aliquots of the peptide stock solution were separated into Eppendorf tubes, frozen, and lyophilized for use in further experiments.

#### 2.3.3. Preparation of Large Unilamellar Vesicles (Liposomes)

Large unilamellar vesicles were prepared using a modified procedure from Wimley [64]. Individual solutions of 1-palmitoyl-2-oleoyl-glycero-3-phosphocholine (POPC) and 1-palmitoyl-2-oleoyl-sn-glycero-3-phospho-(1’-rac-glycerol) (sodium salt) (POPG) lipids (Avanti Research, Alabaster, AL, USA) in chloroform were mixed in a 7:3 mol/mol POPC–POPG ratio, and the chloroform was gently evaporated under a stream of nitrogen gas. The lipid film was then dried *in vacuo* overnight. After complete evaporation of the chloroform, the lipid film was resuspended in the vesicle preparation buffer to yield a 25 mM lipid solution. The vesicle preparation buffer consisted of 45 mM KCl, 10 mM Tris HCl, 8 mM 8-aminonaphthalene-1,3,6-trisulfonic acid disodium salt (ANTS) (Fisher Scientific Inc., Waltham, MA, USA), and 32 mM *p*-xylene-bis-pyridinium bromide (DPX) (Fisher Scientific, Inc.). The lipid film was allowed to dissolve in the vesicle preparation buffer for approximately 15 min. If any lipid film remained on the side of the tube, the solution was gently pipetted up and down to mix the film with the liposome suspension. Once the film was thoroughly dissolved, the lipid suspension was subjected to 10 freeze/thaw cycles using a dry ice/ethanol bath and a 60 °C water bath to facilitate encapsulation of the fluorophore and quencher pair (ANTS and DPX). After 10 rounds of freeze-thawing, the liposomes were extruded through a mini-extruder (Avanti Research) containing a 100 nm polycarbonate membrane to produce liposomes of uniform size. The liposome suspension was passed through the extruder approximately 11 times (or another odd number) to ensure that the liposomes ended in the syringe that had not initially contained the liposome suspension, preventing contamination by larger vesicles. Immediately after extrusion, the liposomes were purified on a column packed with Sephadex^®^ G-100 (Sigma Aldrich, St. Louis, MO, USA) to remove unencapsulated fluorophore, quencher, and free lipids. Before purification, the Sephadex^®^ was pre-equilibrated with liposome elution buffer, containing 75 mM KCl and 20 mM Tris HCl.

#### 2.3.4. Phosphorus Concentration Determination

After purification, the concentration of lipid was determined using a modified Bartlett assay to quantify phosphorus concentration. First, aliquots of a phosphorus standard solution, phosphorus as KH_2_PO_4_, 20 µg/mL in 0.05 N HCl (Sigma Aldrich, cat #P3869), were pipetted into separate test tubes to give samples containing 0, 0.0325, 0.065, 0.114, 0.163, and 0.228 µmoles of phosphorus. Into a separate test tube, 15 µL of the liposome solution was added. Then, to each test tube, 350 µL of 8 M H_2_SO_4_ was added, and the tubes were placed in a test tube heating block at 160 °C for 20 min, after which they were cooled to room temperature. After cooling, 50 µL of a 6% *v/v* solution of hydrogen peroxide was added to each tube, and the tubes were again heated at 160 °C for 40 min, then cooled to room temperature. Following this, 2 mL of water was added to each tube and mixed. A solution containing equal parts 0.28 M ascorbic acid and 10.1 mM ammonium molybdate tetrahydrate was prepared immediately before use, and 0.8 mL of this solution was added to each tube. The tubes were then heated at 105 °C for 7 min. At this point, the tubes developed a blue color, with the intensity of the blue color corresponding to the phosphorus concentration. To prepare the standard curve, 50 µL of the liquid in each tube was mixed with 1.5 mL of water, and the absorbance was measured at 797 nm using a Shimadzu UV-2401 PC Spectrophotometer. The absorbance was plotted against the µmoles of phosphorus, and the absorbance of the liposome sample was used to determine the µmoles of phosphorus. A 1:1 relationship existed between the concentration of phosphorus and the lipid concentration, as each lipid contained one phosphorus atom in its head group. A fresh concentration curve was generated with every new batch of liposomes. An example concentration curve can be found in the Supporting Information (Figure S13).

#### 2.3.5. Preparation of Peptide and Liposomes for Leakage Experiments

Lyophilized peptide was dissolved in the liposome elution buffer at a volume corresponding to half the total volume needed for the experiment. The liposome solution was diluted to 1 mM lipid from the concentration calculated using the phosphorus concentration method described above. The peptide and liposome solutions were then mixed 1:1 to produce the desired peptide concentration and 500 µM lipid.

Fluorescence measurements were conducted on a Tecan Spark^®^ 20M plate reader (Tecan, Männedorf, Zurich, Switzerland). Samples were loaded into a 384-well plate with a transparent bottom. The wells were excited at 355 nm with a bandwidth of 5 nm, and emission was monitored at a wavelength of 520 nm with a bandwidth of 10 nm. The gain and z-position were calculated using a well containing liposomes lysed with reduced Triton^TM^ X-100 (Sigma-Aldrich) to produce maximum fluorescence. The gain was set to be 90% of the optimal gain for wells containing lysed liposomes. Leakage experiments were performed using the optimal gain and z-position for each batch of prepared liposomes.

To ensure accurate comparison across batches of liposomes, the percentage leakage was calculated using the formula:

$$\mathrm{Percent}\;\mathrm{Leakage}=\frac{F-F_{0}}{F_{\mathrm{lysed}}-F_{0}}\times 100$$

where *F* is the relative fluorescence (RF) at time *t*, $F_{0}$ is the RF at t=0, and $F_{\mathrm{lysed}}$ is the RF after the addition of detergent. Since leakage occurred immediately upon mixing the liposome and peptide solutions, the initial fluorescence ($F_{0}$) was determined by adding the relative fluorescence of liposomes alone to the relative fluorescence of the peptide in the elution buffer at each relevant concentration. This approach negated the effect of peptide autofluorescence on the leakage experiments. Leakage experiments were performed at least in triplicate, and error bars were calculated as the standard deviation about the mean.

#### 2.3.6. Circular Dichroism (CD)

Circular dichroism (CD) spectra were recorded using a Jasco-815 circular dichroism spectrometer with a 0.1 mm path-length quartz cuvette. Scans were performed at 25 °C in duplicate, covering the wavelength range of 190–260 nm. The scanning parameters included a 1.0 nm data pitch, 1.0 nm bandwidth, and a scanning speed of 100 nm/min.

Background correction was applied to the spectra: for samples without liposomes, the spectra of the buffer alone were subtracted, and for samples containing liposomes, the spectra of liposomes in the buffer were subtracted. Ellipticity (Θ) was converted into molar ellipticity ([Θ]) using the formula:

$$\left[\Theta \right]=\frac{\Theta }{10\cdot c\cdot l}$$

where [Θ] has units of deg cm^2^ dmol^−1^, Θ is the measured ellipticity in degrees, *c* is the molar concentration of the peptide, and *l* is the path length in cm.

#### 2.3.7. Fourier Transform Infrared Spectroscopy (FT-IR)

FT-IR spectra were obtained on a Perkin Elmer Spectrum 3. Before analysis, samples were subjected to two rounds of D_2_O/DCl exchange to remove TFA counter-ions by first dissolving the peptide in 40% MeCN/D_2_O with 1% DCl (*v/v*) and freezing and lyophilizing, and then dissolving in a 0.1% DCl solution and freezing and lyophilizing. After both rounds of exchange, the peptide was dissolved in liposome elution buffer that had similarly been D_2_O exchanged, and finally dissolved in D_2_O. Samples were injected into a 25 mm × 4 mm CaF_2_ plate (International Crystal Labs), and spectra were obtained using the Happ–Genzel method from 1550 to 1750 cm^−1^ with a 2 cm^−1^ resolution (8 scans).

#### 2.3.8. Cryo-Electron Microscopy (Cryo-EM)

To prepare samples for cryo-electron microscopy (cryo-EM), c-flat copper grids were glow-discharged for 30 s at 30 mA using room air. A 3 µL aliquot of the sample was vitrified on the glow-discharged grids at room temperature in 95% humidity, with a 3.5 s sensor blot, using a Leica EM-GP2 plunge freezer. Samples were imaged using low-dose imaging on a Talos 120C electron microscope, equipped with TIA software, to minimize electron beam damage during imaging.

## 3. Results

### 3.1. Water Permeation

All-atom simulations of peptide–membrane systems were performed to assess the potential of our model peptides to disrupt the integrity of lipid bilayers. Figure 2 shows the cumulative number of water permeation events over time, with dashed lines representing control simulations without peptides. Figure 2A presents the results for the 8-residue peptides M01 [Ac-(FKFE)_2_-NH_2_] and M02 [Ac-FFKKFFEE-NH_2_], which preferentially adopted β-sheet and α-helical conformations, respectively, at the water–lipid interfaces [28]. In these simulations, 10 peptides were added into the solution every 1 µs, which allowed for their gradual adsorption and aggregation on the membrane. This corresponds to an insertion of 80 amino acids at each step–Figures S1 and S2. M01 peptides adsorb to the membrane and progressively form β-sheet-rich aggregates. Dimers and trimers appeared in simulations containing 20–30 peptides, and larger assemblies—tetramers and pentamers—emerged after the insertion of an additional 10 peptides (totaling 40)–Figure S1. These clusters continued to grow as the simulation progressed and they eventually penetrated the bilayer. Membrane insertion occurred when β-sheets on opposing leaflets aligned to form a transmembrane water channel. Additional β-sheets were recruited to form a cylindrical structura around the pore, with nonpolar side chains oriented toward the lipid core and polar residues lining the aqueous interior. This structural rearrangement explains the sharp rise in water permeation at 5 µs–cyan line in Figure 2A. Panel I shows the resulting pore formed by M01, with the largest β-sheets composed of up to 10 peptides spanning the membrane.

In simulations with peptide M02, the cumulative water permeation curve (purple line) showed no sharp increase indicative of pore formation (Figure 2A). The molecular structure of our system at the end of the simulation shows that the 40 adsorbed peptides were dispersed on the membrane leaflets folded into helical structures–panel II. The latter conformation partitions polar and nonpolar residues to the solvent and membrane interior, respectively, and it leaves only a reduced number of dangling peptide hydrogen donors and acceptors–Figure 1. In contrast, M01 adopts extended β-strand conformations, driven by its alternating polar–nonpolar sequence pattern. This arrangement leaves many hydrogen donor and acceptor sites exposed, which is energetically unfavorable unless passivated—explaining the spontaneous β-sheet formation observed in Figure 2B.

The picture that emerges from Figure 2A is that M01 peptides are able to create pores due to their ability to form large β-sheet aggregates that can span both membrane leaflets. This contrasts with the tendency of M02 peptides to remain dispersed on the lipid membrane. In addition, individually, M02 peptides are too short to penetrate deeply within the bilayer and cause damage. These insights inspired us to study two longer 16-residue peptides that can span the bilayer when folded into an α-helix. The first peptide sequence, Ac-(FFKKFFEE)_2_-NH_2_ (M03), corresponds to the juxtaposition of two M02 repeats. As shown in Figure 1, this sequence produces α-helices with one face that is mostly (but not completely) non-polar and the other polar. The sequence of the second studied peptide named M04 is Ac-(FFKKFFEE)(FKKFFEEF)-NH_2_ and corresponds to the juxtaposition of the M02 sequence with one of its 8-residue permutations. This combination is designed to allow for perfect segregation of polar–nonpolar residues to different faces of an α-helix—Figure 1. Thus, both peptides contain the same sixteen amino acids and share the same N-terminal sequence, beginning with two nonpolar residues. They differ in their ability to segregate polar and non-polar residues, and in their last C-terminal residue, which is negatively charged in M03 and nonpolar in M04.

Figure 2B shows the number of water permeation events in three independent simulations performed using M03 (gray-black lines) and M04 (pink-red) peptides. In these simulations, five peptides were added to the system every 1 µs, accounting for the same amino acid concentration of added peptides as in panel A. All three simulations performed with the M03 peptide resulted in pore formation characterized by a large increase in the number of water permeation events at ∼2.5 µs and ∼4.5 µs. The pore from the simulation represented by the black line in panel B is depicted in panel III, showing peptide accumulation in the form of a cylindrical channel that spans both leaflets of the membrane. This channel is filled with water in its interior. In stark contrast, simulations performed with M04 peptides did not account for a sudden increase in water permeation events. Accordingly, we found that the peptides, at the end of the simulation (panel IV), were mostly dispersed on the membrane, without piercing it. Thus, despite the *perfect* segregation of polar–nonpolar residues to different faces in an α-helix, which is a feature of many membrane active peptides, the M04 sequence has a significantly lower potential to damage lipid membranes than M03. Notice, however, that in one of the M04 simulations (red line), the number of water permeation increased by a small amount at time ∼2.5 µs. This suggests that M04 peptides also have the potential to damage lipid membranes, although much less than M03.

### 3.2. Mechanism of Membrane Damage

**Figure 3:**
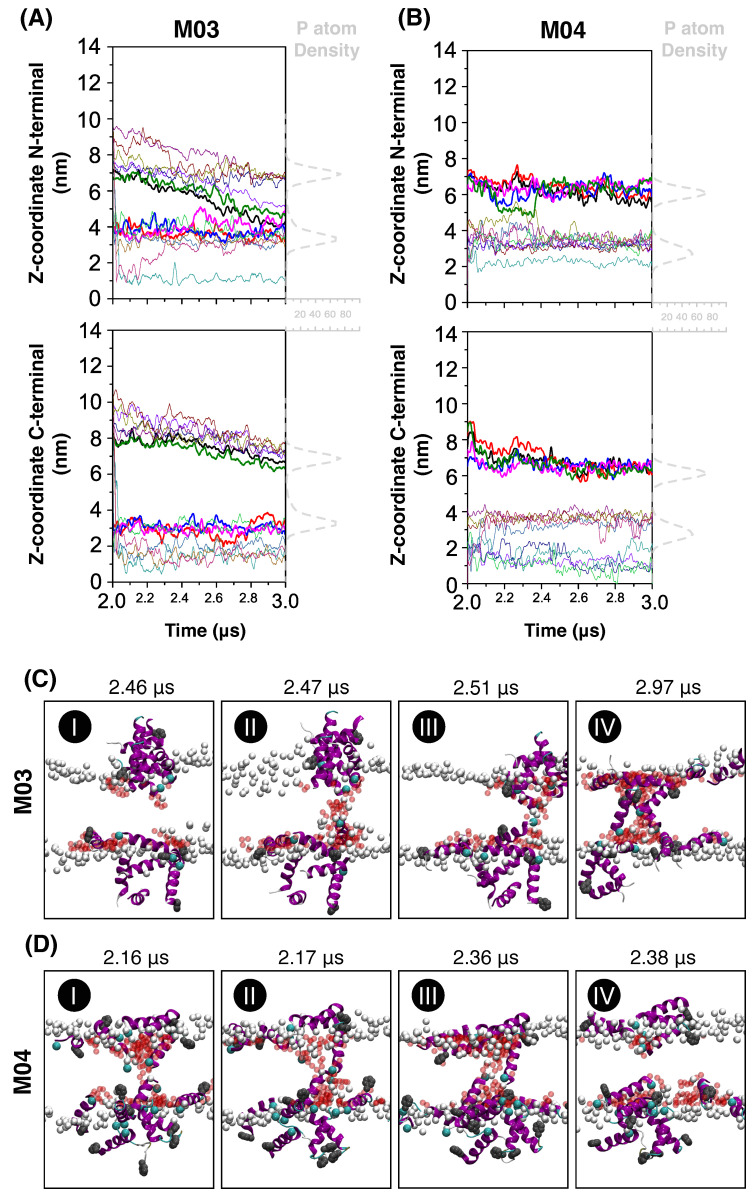
(**A**,**B**) Positions of N-termini (top panels) and C-termini (bottom panels) for each peptide in simulations from Figure 2B: M03 (black) and M04 (red). Dashed gray lines correspond to the density peaks of phosphate atoms. (**C**,**D**) Representative molecular structures from simulations with M03 and M04 peptides during membrane disruption. Phosphate atoms are shown as gray spheres; N- and C-termini are marked by cyan and dark-gray spheres, respectively. Red beads indicate water oxygen atoms inside the bilayer.

To investigate how peptide–lipid interactions at the bilayer surface lead to pore formation and water leakage, Figure 3A,B track the z-coordinates of the N- and C-termini of each peptide during the simulations. This analysis focuses on the 2–3 µs interval, when water permeation begins to occur for both peptides—Figure 2B. The results show that both M03 and M04 peptides inserted into the membrane via their N-termini (upper panels), while their C-termini (lower panels) remained anchored at the membrane surface throughout the simulation. This behavior was initially surprising, given that the first eight residues are identical in both sequences, yet the extent of their water leakage differed substantially. Figure 3 also shows that, in the M03 system, three peptides (black, green, and purple lines) from the upper leaflet and one (pink) from the lower leaflet penetrated the membrane after 2.4 µs. In contrast, intermittent leakage in the M04 system arose between 2.2 and 2.4 µs, as one peptide from each leaflet (green and brown lines) converged near the center of the bilayer.

Figure 3C,D illustrate the sequence of events leading to pore formation by M03 and M04 peptides. The deposition of small aggregates on the membrane surface (panels C-I and D-I) indicates that peptides can self-assemble in solution before interacting with lipids. As the simulation progressed, some of these peptide–peptide interactions were exchanged for peptide–lipid interactions. Pore formation starts when water molecules (red spheres) surrounding the N-termini (cyan spheres) of peptides in opposing leaflets converge at the bilayer center–C-II and D-II. This coalescence forms a continuous water column across the membrane, which enables the polar face of the helices to stabilize in the non-polar environment of the bilayer–C-IV and D-III. This pore remained stable until the end of the simulation for M03 peptides. In contrast, the pore was only metastable for M04 peptides and the membrane healed itself within 0.2 µs (panel D-IV), expelling peptides to its surface.

**Figure 4:**
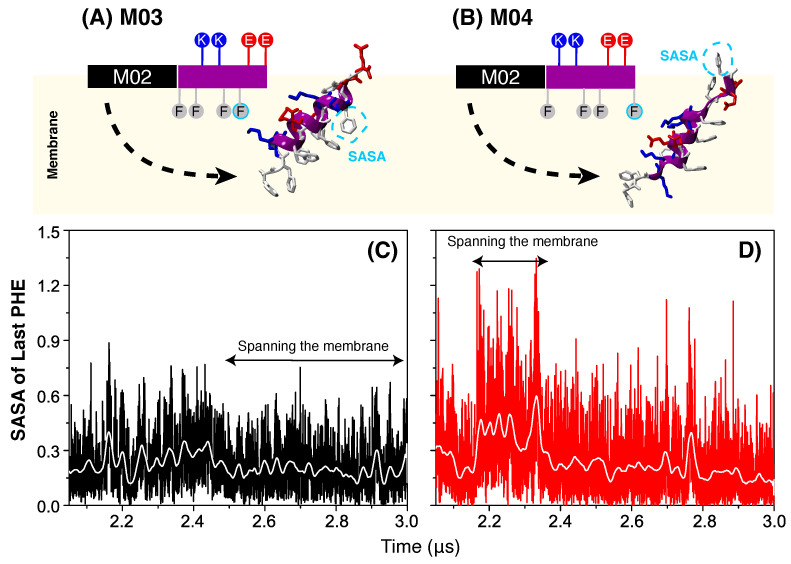
Solvent exposure of the C-terminal. (**A**,**B**) Schematics of M03 and M04 peptides with their N-termini aligned parallel and inserted into the membrane. (**C**,**D**) Solvent-accessible surface area (SASA) of the last phenylalanine of the M03 and M04 peptides that are inserted in the membrane. A 15-point moving average (white line) is shown for clarity. Horizontal arrows indicate time intervals when the N-terminal remained embedded in the membrane.

To understand why insertions of M03 peptides are more stable those of M04, Figure 4 examines the interaction of their C-termini with the lipid bilayer. Specifically, we focus on the terminal nonpolar residue, which is positioned closer to the C-terminus in M04 than in M03 (Figure 4A,B). When peptides rotate to insert their N-termini into the membrane, this nonpolar residue can become exposed to the aqueous environment—an energetically unfavorable configuration. We hypothesize that such exposure is more pronounced for M04, potentially explaining its reduced membrane-disrupting ability. To test this, we computed the solvent-accessible surface area (SASA) of the terminal phenylalanine, averaged over peptides that penetrated the membrane. For the M03 peptides, which become inserted into the membrane after 2.4 µs, the SASA remained relatively constant throughout the simulation—Figure 4C. In contrast, the SASA of the M04 peptides that inserted between 2.2 and 2.4 µs increased significantly, indicating greater exposure of the non-polar residues to water. This unfavorable interaction likely contributed to the reduced pore stability and membrane disruption observed for M04.

### 3.3. Experimental Investigation

It is important to highlight that all-atom simulations were performed starting with M03 and M04 peptides already folded into α-helices. This was motivated by simulations of M02 peptides that, starting from random coil conformations, spontaneously folded into α-helices at the water–lipid interface–Figure 2-II. An α-helix conformation for M03 and M04 peptides was also expected, based on the segregation of polar and nonpolar residues to different faces of a helical wheel—Figure 1. However, since α-helix conformations were critical for pore formation in the simulations, the ability of M03 and M04 peptides to form this secondary structure needed to be tested experimentally. In addition, the larger permeation of M03 compared to M04 in the simulations was unexpected, since sequences with higher hydrophobic moments (µ) are usually associated with more intensive water leakage [41], whereas M03 (μ = 0.59) had a smaller moment than M04 (μ = 0.87) [47]. Here, experiments were performed to test this water leakage trend.

In the following section, we discuss the secondary structure and membrane damage properties of M03 and M04. While modeling shows that M01 is capable of damaging membranes, experimentally the peptide self-assembled into supramolecular β-sheet rich fibers [27,65]. This happened immediately upon dissolution in aqueous solvent; likely before it was able to interact with the membranes. Therefore, it was difficult to determine the effect of M01 peptides on membranes experimentally, as the leakage observed was likely due to mechanical disruption of the liposomes as the peptide monomers assembled into their final fibril structure. While modeling of M02 suggests that it forms an α-helix, experimentally, the analysis of its secondary structure was less straightforward. The IR spectra (Figure S3) contain peaks that overlap with both the random coil and α-helical region, indicating that more complex methods of secondary structure determination are needed to experimentally validate the models. Despite this ambiguity, the leakage assays of M02 were consistent with the modeling and showed minimal leakage, even at high concentrations of peptide (Figure S4).

Notice that the experiments and simulations were performed under different conditions. The former used room temperature and a buffer salt solution, whereas the simulations were performed at a higher temperature of 350 K and in pure water. The latter temperature was necessary to accelerate the dynamics enabling pores to form within a few microseconds of simulation. The addition of salts to the solution was expected to accelerate peptide aggregation, as well as to affect secondary structure formation, and peptide adsorption onto lipid membranes [20,25,66,67,68,69]. Direct comparison between the simulations and experiments is therefore not possible. However, the ranking of peptide sequences based on their ability to form pores is robust across a broad range of conditions/solutions. This allows in silico and in vitro studies to inform each other and will be explored here.

#### 3.3.1. Secondary Structure

**Figure 5:**
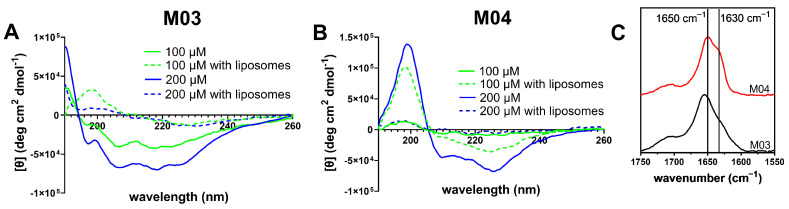
CD spectra in liposome elution buffer of (**A**) M03 and (**B**) M04 at various peptide concentrations, with and without liposomes. Lipid concentration was 500 µM for all experiments containing liposomes. (**C**) FT-IR of M03 and M04 at 500 µM.

To investigate the secondary structure of the peptides modeled above, circular dichroism (CD) and Fourier transform infrared (FT-IR) spectra of each peptide sequence were obtained and analyzed. Both CD and FT-IR spectra were recorded immediately after dissolving the peptides in the liposome elution buffer. For the experimental details, please refer to the Methodology section. For CD, additional spectra were recorded in the presence of large unilamellar vesicles (LUVs). The CD spectra (Figure 5A,B) indicate that both peptides adopted at least a partial α-helical secondary structure, as evidenced by minima at 208 nm and 222 nm [70]. Peptide M03 also exhibited a positive peak at 190 nm, another characteristic feature of an α-helix. However, in addition to the typical α-helical peaks, both peptides showed spectral features consistent with β-sheet structures, such as minima at 218 nm. M04 also displayed a maximum at 197 nm, indicative of a β-sheet character. These results suggest that the secondary structure of each peptide was a complex mixture of α-helical and β-sheet content.

Additionally, the spectra recorded in the presence of the LUVs differed from those recorded in buffer alone. Peptide M03 showed an increase in CD signal as the peptide concentration increased from 100 µM to 200 µM in the absence of LUVs (Figure 5A). However, spectra recorded in the presence of lipid vesicles showed a marked decrease in signal at both concentrations, likely due to aggregation of the peptides as they interacted with the lipid membrane. At peptide concentrations higher than 500 µM, M03 began to lose solubility, while M04 began to precipitate from solution at concentrations higher than 200 µM. The reduced solubility of M04 was likely due to the orientation of all its hydrophobic residues on one face of the α-helix, making it more prone to aggregation in aqueous solutions. Interestingly, M04 in the absence of liposomes also showed an increase in CD signal at 200 µM compared to 100 µM. At a concentration of 100 µM, the CD signal was stronger in the presence of the liposomes than in their absence, suggesting that interaction with the lipid membrane may have induced the formation of a secondary structure in M04, a common characteristic of membrane-active peptides [71,72,73]. It is also important to note that the CD spectra of M03 and M04 were noticeably different from each other. This underscores the effect that varying the sequence pattern, even slightly, can have on the resulting peptide properties.

While CD spectroscopy is a classic technique for probing secondary structures, its analysis can become complicated, especially in peptides and proteins containing aromatic residues [70]. Therefore, Fourier transform infrared (FT-IR) spectroscopy can also provide valuable insights into peptide secondary structure by analyzing shifts in the absorbance of the amide I stretch. Peptides with α-helical structures typically exhibit absorbance between 1655 and 1638 cm^−1^, while those with β-sheet character absorb around 1630 cm^−1^ in D_2_O [74]. Unordered structures are indicated by absorbance at approximately 1650 cm^−1^. Both peptides M03 and M04 displayed peaks near 1650 cm^−1^ and 1630 cm^−1^ (Figure 5C). While the peak at 1650 cm^−1^ could represent either disordered or α-helical structures, the FT-IR spectra, in conjunction with the CD results, suggest an α-helical orientation is more likely. However, the presence of β-sheet indicators in both CD and FT-IR spectra suggests that the peptides adopt a mixture of α-helical and β-sheet structures in solution. These findings demonstrate a more complex secondary structure than predicted by modeling, highlighting the interplay of experimental and computational methods in elucidating peptide behavior.

#### 3.3.2. Leakage Assays

A modified leakage assay, in which a fluorophore and quencher pair were encapsulated within the liposomes [64], was used to experimentally probe the effect of M03 and M04 peptides on model membranes, to compare with the computational models. In the assay, large unilamellar vesicles (LUVs) were made using a 7:3 ratio of POPC–POPG lipids, giving the membranes an overall negative charge due to the negatively charged POPG lipids, which could facilitate electrostatic interactions between the membrane and the positively charged amino acid residues of the peptides. To confirm that the vesicles were unilamellar after extrusion, cryo-EM images of the liposome solution were obtained, revealing that approximately 90% of the solution contained uniform, unilamellar vesicles (Figure S4). A fluorophore and quencher pair (ANTS/DPX) were encapsulated within the interior of the vesicles, leading to quenching of the fluorophore due to their close proximity. Damage to the membrane was assessed by measuring the percent leakage of the fluorophore and quencher from the interior of the vesicles. Percent leakage was calculated from the increase in fluorescence observed as the fluorophore/quencher pair diffused away from each other in free space after escaping the confines of the LUVs. Maximum release, or maximum fluorescence, was determined by adding reduced Triton™ X-100 detergent to the samples. Percent leakage was calculated using the following equation:

(1) $$\mathrm{Percent}\;\mathrm{Leakage}=\frac{F-F_{0}}{F_{\mathrm{lysed}}-F_{0}}\times 100,$$

where *F* is the relative fluorescence (RF) at time *t*, $F_{0}$ is the RF at t=0, and $F_{\mathrm{lysed}}$ is the RF after the addition of the detergent.

**Figure 6:**
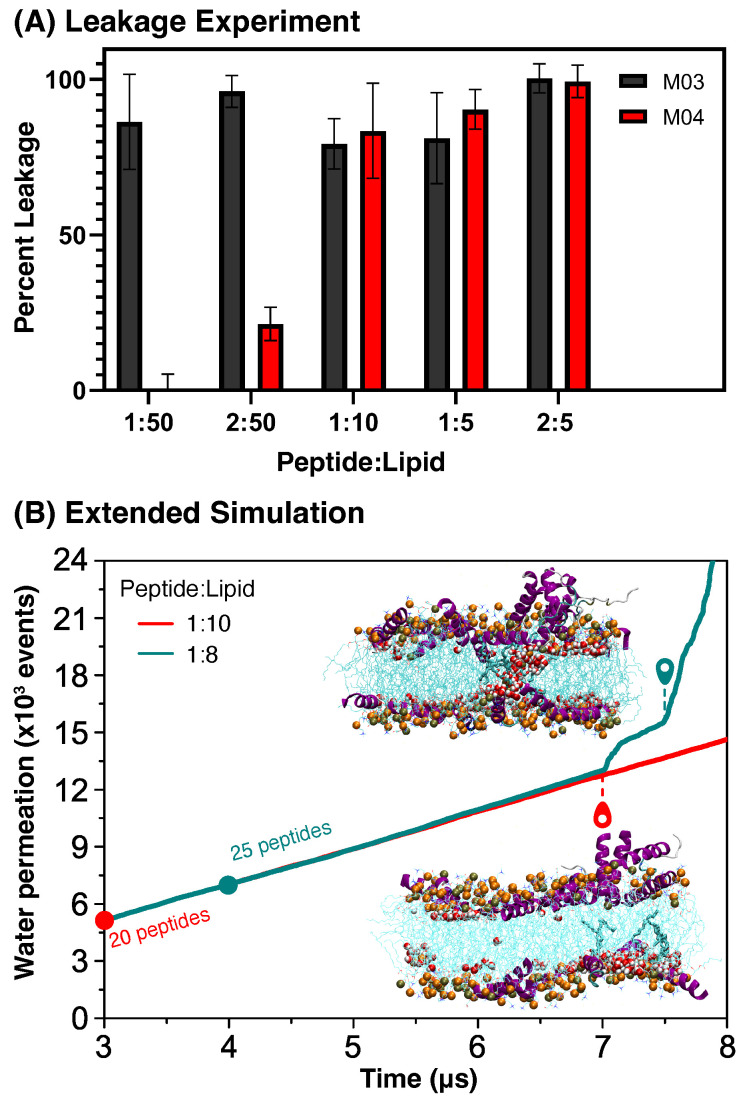
Membrane disruption and water leakage. (**A**) Leakage assay quantifying water permeation induced by M03 (black) and M04 (red) peptides at different peptide-to-lipid (P:L) mass ratios. (**B**) Cumulative water permeation events from MD simulations with 20 (red) and 25 (cyan) M04 peptides. Cross-sectional views of the membrane are shown for simulations with 25 (top) and 20 (bottom) peptides.

Leakage experiments for peptides M03 and M04 were performed at peptide–lipid (P:L) ratios of 1:50, 2:50, 1:10, 1:5, and 2:5, and the results of these leakage studies are shown in Figure 6A. The percentage of leakage was greater than 80% at all P:L ratios for M03 peptides, suggesting that it can damage lipid membranes even at low concentrations. In contrast, there was no leakage at the lowest ratio (1:50) studied for M04 peptides, and at the second lowest ratio (2:50), the percentage of damage was only 21%. For the ratios tested, the P:L ratio had to be at least 1:10 for M04 peptides to produce a level of leakage that was comparable to M03 at any of the ratios. This corroborates the results from the MD simulations, showing that M03 peptides exhibited a higher tendency to damage lipid membranes than M04. In addition, these experiments suggest that, if simulations of M04 peptides are performed at higher concentrations, they can cause significant membrane damage.

#### 3.3.3. High Concentration

In our simulations with up to 20 peptides, M04 did not form pore-like structures inside lipid membranes (Figure 2B), although the experiments in Figure 6A show water leakage at high concentrations. To test the consistency of MD models with the latter experiments, additional simulations were performed with 25 × M04 peptides for 8 µs. The accumulated number of water permeation events in this simulation is shown in cyan in Figure 6B, and this is compared with simulations performed with 20 peptides—red line reproduced from Figure 2B. At the time 7 µs, water abruptly permeated the membrane in the simulation with 25 × M04 peptides, as a pore-like structure was formed. The latter was surrounded by a high concentration of peptides that remained on the surface of both leaflets of the bilayer, without penetrating its dry core. By remaining on the surface, M04 peptides do not expose their last phenylalanine to water, as in Figure 4B. Thus, the membrane damage by M04 peptides at high concentration could also be modeled using MD simulations, although significant computational resources would be required for that purpose.

## 4. Conclusions

In summary, this paper compared the lipid membrane damage by four neutral amphipathic peptides. Two of these peptides, named M01 and M02, are 8-residue isomers made using four phenylalanines, two glutamic acids, and two lysines. At the water–lipid interface, peptides M01 and M02 fold into β-strands and α-helices, respectively. In unbiased all-atom simulations, M01 peptides were found to aggregate promptly into long β-sheets that spanned both leaflets of the bilayer, allowing water to permeate the membrane. In contrast, α-helical M02 peptides remained dispersed on the membrane surface, without spanning its leaflets or causing water leakage. To provide insights into the potential of α-helices to damage lipid membranes, two longer (16 residues) versions of the M02 peptide, named M03 and M04, were studied.

The M03 peptide is made by repeating the M02 sequence twice. This results in a peptide with μ=0.57, and N- and C-termini that are nonpolar and charged, respectively. The M04 peptide is made by moving one of the non-polar amino acids in the middle of the M03 sequence to the C-terminal. The latter modification accounts for a sequence in which non-polar and charged amino acids are more sharply segregated at the water–lipid interface (μ=0.87), and with both N- and C-terminals exhibiting non-polar residues. In leakage experiments, this modification increased the minimal peptide concentration required to induce water permeation by at least a factor of 5. The peptide concentration was less than 1:50 peptide–lipid for M03 peptides and 1:10 for M04. This difference in behavior in the all-atom simulations is explained by the higher propensity of M03 peptides to insert themselves into the bilayer spanning its leaflets. This starts with peptides parallel to the membrane rotating around charged residues in the C-terminal, which behave as a pivot point. In this process, water molecules are dragged to the interior of the bilayer. In contrast, rotation is mostly inhibited for M04 peptides, as non-polar residues close to the pivot point at the C-terminal become partially exposed to the solvent when M04 helices rotate to span the bilayer. Thus, in the computer simulations and experiments, M04 peptides only induced water permeation at higher concentrations compared to M03.

Taken together, our results highlight properties in the amino acid sequence that account for membrane damage by amphipathic peptides. These include partitioning of polar–nonpolar residues at the water–lipid interface, which determines the secondary structure of peptides; the length of these secondary structures compared to the bilayer thickness; and the difference in hydropathy between the N- and C-terminals. Consistent with other studies, we found that peptides that form secondary structures with end-to-end distance of ∼2 nm or more can induce water permeation [35,36,37]. This is the case for the 8-residue β-strand (M01) and 16-residue α-helical (M03 and M04) peptides studied here, but not for the 8-residue α-helical M02 peptide. Compared to M03, the M04 peptide could have been expected to be more efficient at inducing water permeation, since its amphiphilicity is enhanced. However, our simulations showed that its C-terminal non-polar residue protrudes from the membrane as its N-terminus inserts into the bilayer, causing it to become hydrated and less prone to forming pores. Thus, although our study confirms that amphiphilicity is important in accounting for secondary structure formation, other aspects of the amino acid sequence, e.g., differences in the hydropathy of N- and C-terminals, can modulate the ability of a peptide to damage the membrane via pore formation. We anticipate that some of the effects of C-terminal modifications reported in the literature [42,43] may be explained by this difference, which should be further explored as a design principle.

It is important to highlight that although membrane damage is a stochastic process that involves aggregation within the lipid bilayer, the results from our simulations are highly consistent. Leakage was observed in three independent simulations performed using M03 peptides, but in none of the three simulations performed with M04 peptides. Membrane damage of the latter peptides was only observed at a higher concentration, in agreement with the experiments. However, this consistency can only be obtained if simulations are performed for very long times, i.e., ∼10 µs. Accordingly, the increased accessibility of computers that can reach these timescales is likely to expand the use of all-atom simulations to new interesting problems. This will require a calibration of force fields to ensure the right balance between peptide–peptide, peptide–lipid, and lipid–lipid interactions.

## Data Availability

The data presented in this study are available on request from the corresponding author.

## Supplementary Materials

The following supporting information can be downloaded at: https://www.mdpi.com/article/10.3390/biom15070973/s1, Figure S1: Molecular structure of the peptide-membrane system for the 8-residue sequences M01 and M02. Lipids are depicted in gray, and a color scheme is used to represent secondary structures. Yellow is used for β-strands, purple and blue for α- and π-helices, and cyan for turn/bend; Figure S2: Molecular structure of the peptide-membrane system for the 16-residue sequences M03 and M04. Lipids are depicted in gray, and a color scheme is used to represent secondary structures. Purple and blue are used for α- and π-helices, and cyan for turn/bend; Figure S3: Experimental leakage data of M02 with LUVs. Leakage experiments were performed at a lipid concentration of 500 μM and at peptide concentrations of 100 μM, 500 μM, and 1 mM, corresponding to peptide:lipid ratios of 1:5, 1:1, and 2:1, respectively; Figure S4: Cryo-EM image of a sample of the LUVs produced and used in these experiments. Grids were flash frozen immediately after preparation of the vesicles and scale bar is 200 nm; Figure S5: MALDI-TOF spectra M02; Figure S6: MALDI-TOF spectra M03; Figure S7: MALDI-TOF spectra M04; Figure S8: Analytical HPLC trace M02; Figure S9: Analytical HPLC trace of M03; Figure S10: Analytical HPLC trace of M04; Figure S11: Representative analytical HPLC concentration curve of M02; Figure S12: Representative analytical HPLC concentration curve for M03 and M04; Figure S13: Representative phosphorus concentration curve; Table S1: Preparatory scale HPLC gradients for purification; Table S2: Calculated and observed m/z for all peptides by MALDI-TOF-MS; Table S3: Analytical HPLC gradient conditions and peptide retention times.
