# Supplementary material for: Insights into Membrane Damage by α-Helical and β-Sheet Peptides
Source: Biomolecules. 2025 Jul 7;15(7):973. doi: 10.3390/biom15070973 (PMC12292791; doi:10.3390/biom15070973)
Supplement: Supplementary file 1 [file biomolecules-15-00973-s001.zip › biomolecules-3703925-supplementary.pdf]

Insights into membrane damage by α-helical and β-sheet peptides.

Warin Rangubpit[§†], Hannah E. Distaffen[§‡], Bradley L. Nilsson[‡¶*], and Cristiano L. Dias[*†]

[†]Department of Physics, New Jersey Institute of Technology, Newark, New Jersey, 07102-1982 USA

[‡]Department of Chemistry, University of Rochester, Rochester, New York, Rochester, NY 14627-0216

[¶]Materials Science Program, University of Rochester, Rochester, New York 14627-0166

* Corresponding Authors: E-mail: cld@njit.edu and bradley.nilsson@rochester.edu

1. Molecular structure of peptides in all-atom simulations

In simulations, peptides are introduced into the solution every 1 µs, allowing spontaneous adsorption and aggregation on the membrane. For the 8-residue sequences M01 and M02, 10 peptides (or 80 amino acids) are added at each insertion step. For the 16-residue sequences M03 and M04, peptide insertion was performed at the same amino acid concentration, which corresponds to 5 additional peptides at every step. Figures S1 and S2 show the molecular structure of the peptide-membrane system at the end of each step.

At the end of each insertion step, we observe aggregation progressing for M01 and M03 peptides. For M01 peptides, dimers and trimers are observed in simulations containing 160 and 240 amino acids—Fig. S1. Some of these small clusters increase in size to form tetramers and pentamers after another 10 peptides are embedded into the solution (320 amino acids) and simulated for 1 µs. For M03 peptides, tetramers form already in simulations containing 10 peptides (i.e., 160 aa). In simulations containing 15 peptides (240 aa), tetramers and pentamers are observed on both leaflets. All peptides are part of the aggregates in upper or lower leaflets at the end of the simulation containing 20 peptides (320 aa). In contrast, peptides M02 and M04 do not aggregate significantly.

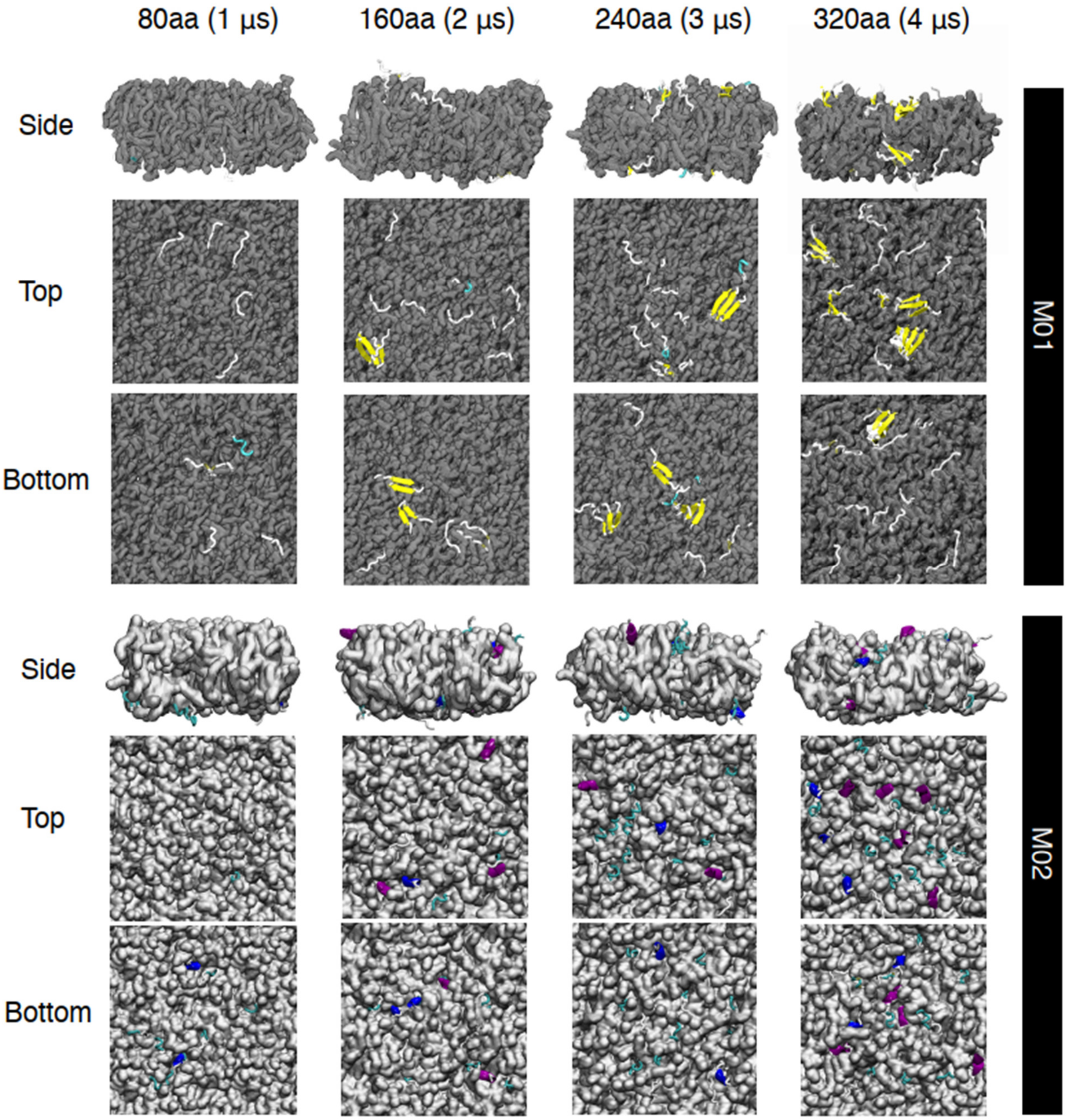

Figure S1. Molecular structure of the peptide-membrane system for the 8-residue sequences M01 and M02. Lipids are depicted in gray, and a color scheme is used to represent secondary structures. Yellow is used for β-strands, purple and blue for α- and π-helices, and cyan for turn/bend.

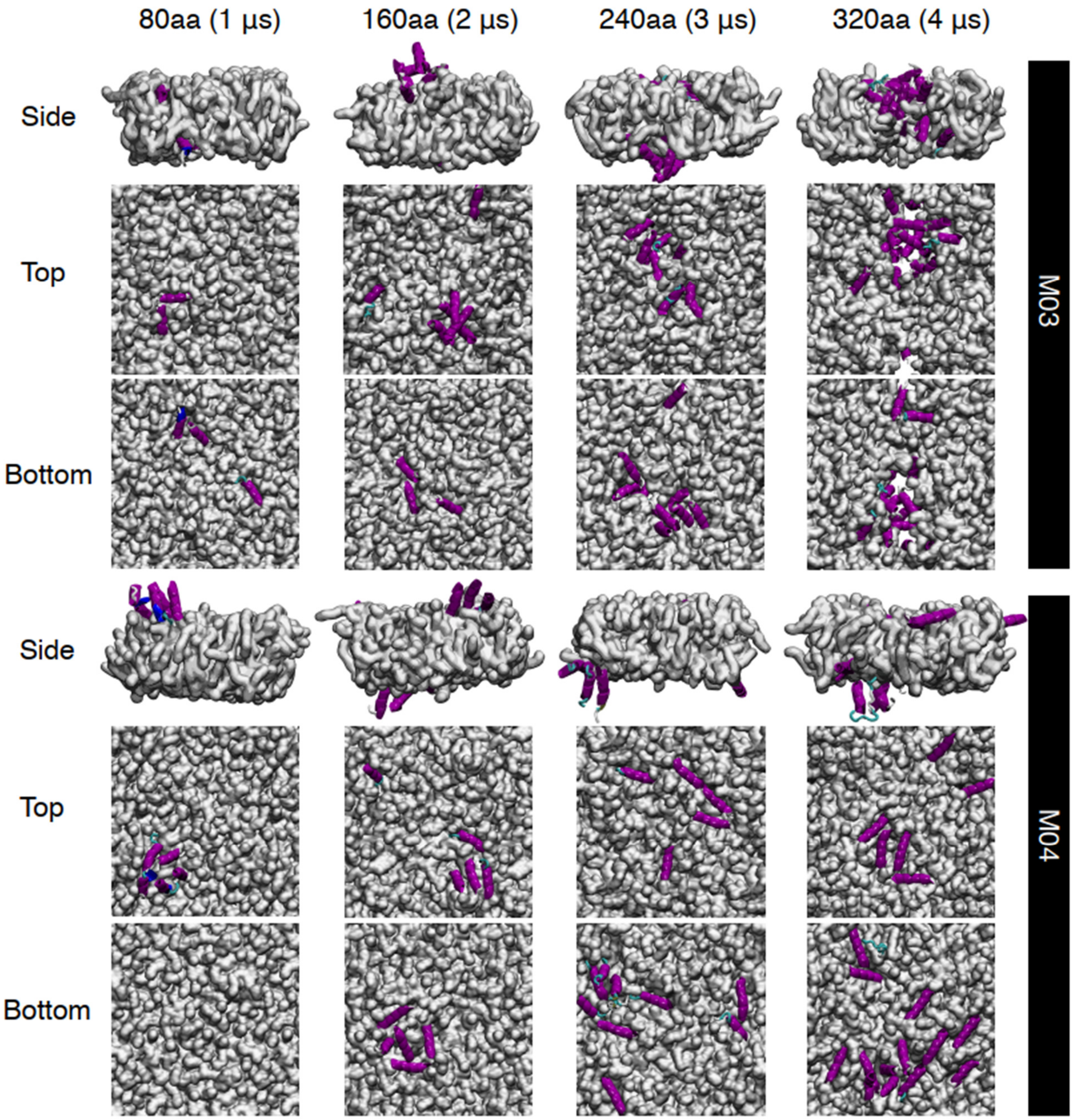

Fig. S2. Molecular structure of the peptide-membrane system for the 16-residue sequences M03 and M04. Lipids are depicted in gray, and a color scheme is used to represent secondary structures. Purple and blue are used for α- and π-helices, and cyan for turn/bend.

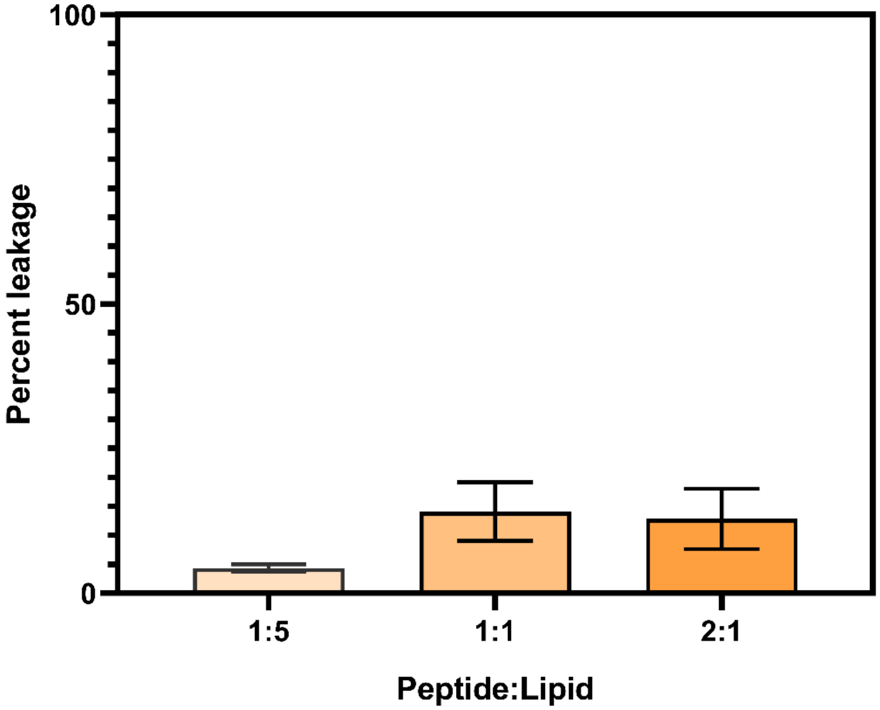

Figure S3. Experimental leakage data of M02 with LUVs. Leakage experiments were performed at a lipid concentration of 500 µM and at peptide concentrations of 100 µM, 500 µM, and 1 mM, corresponding to peptide:lipid ratios of 1:5, 1:1, and 2:1, respectively.

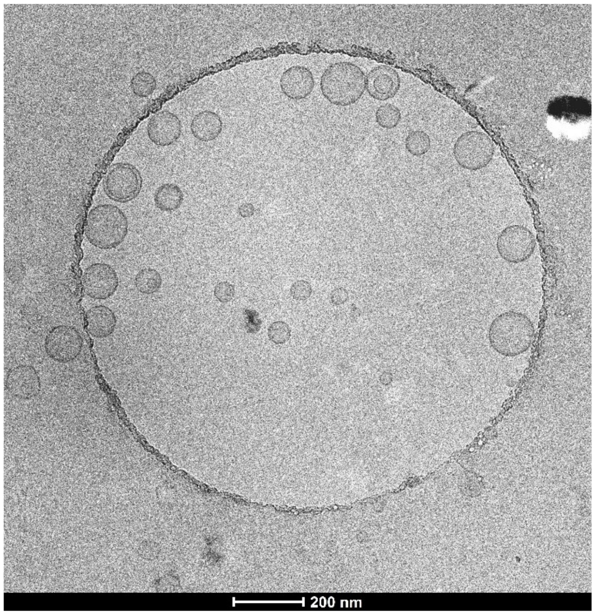

Figure S4. Cryo-EM image of a sample of the LUVs produced and used in these experiments. Grids were flash frozen immediately after preparation of the vesicles and scale bar is 200 nm.

Table S1. Preparatory scale HPLC gradients for purification.

**Peptide**	**Sequence**	**Retention Time (min)**	**Gradient (Soln A:** water/0.1% TFA, **Soln B:** acetonitrile/0.1% TFA)
**M02**	Ac-FFKKFFEE-NH_2	18.54	22-30% **B** over 15 minutes, 95% **B** over 5 minutes, 22% **B** for 5 minutes. Flow rate 50 mL/min
**M03**	Ac-$(FFKKFFEE)_2$-NH_2	6.40	28-32% **B** over 12 minutes, 95% **B** for 5 minutes, 28% **B** for 5 minutes. Flow rate 100 mL/min
**M04**	Ac-FFKKFFEEFKKFFEEF-NH_2	9.00	27-30% **B** over 12 minutes, 95% **B** for 5 minutes, 27% **B** for 5 minutes. Flow rate 100 mL/min

Table S2. Calculated and observed *m/z* for all peptides by MALDI-TOF-MS.

Peptide	Calc. $[MH^+]$ *m/z*	Obs. $[MH^+]$ *m/z*	Calc. $[MNa^+]$ *m/z*	Obs. $[MNa^+]$ *m/z*	Calc. $[MK^+]$ *m/z*	Obs. $[MK^+]$
M02	1162.6	1164.2	1184.6	1186.9	1200.7	1202.7
M03	2265.12	2264.8	2287.13	2286.3	2303.24	2302.4
M04	2265.12	2264.6	2287.13	-	2303.24	-

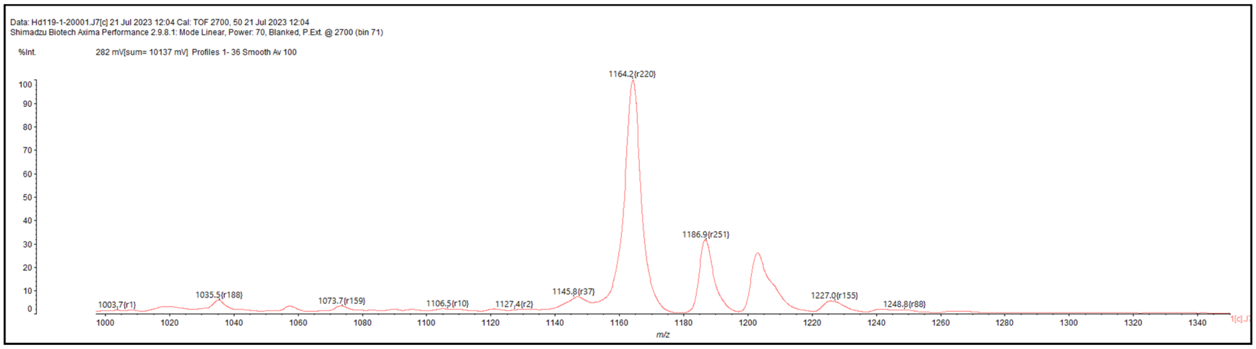

Figure S5. MALDI-TOF spectra M02.

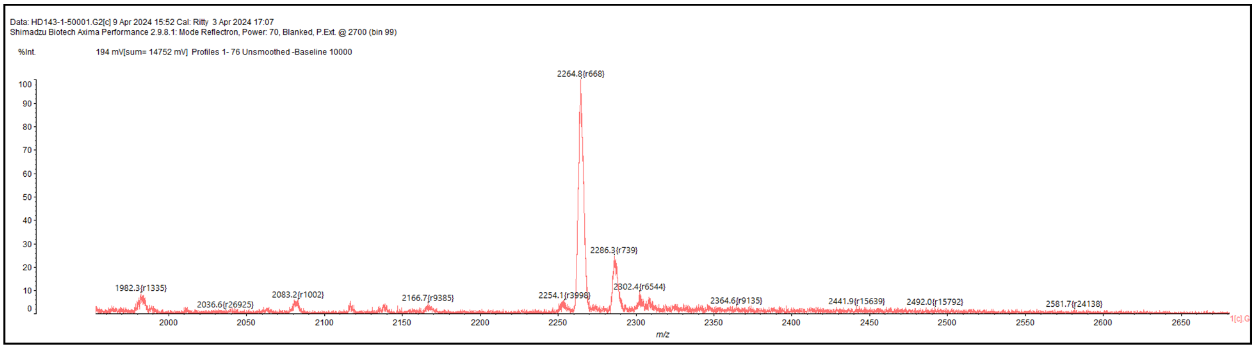

Figure S6. MALDI-TOF spectra M03.

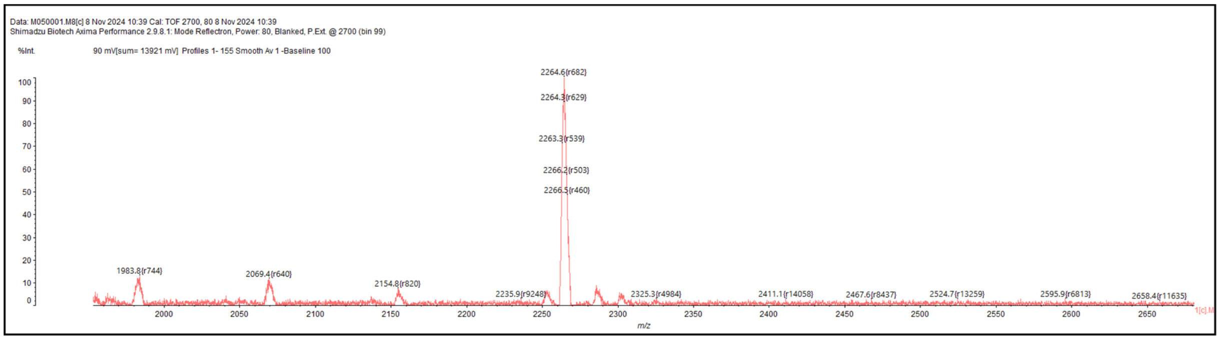

Figure S7. MALDI-TOF spectra M04.

Table S3. Analytical HPLC gradient conditions and peptide retention times.

Peptide	Retention Time	Gradient (Soln A: water/0.05% TFA, **Soln B:** acetonitrile/0.05% TFA)
M02	12.45	Isocratic 5% **B** 5 min, 5-95% **B** over 10 min, 95% **B** 5 min
M03	13.11	Isocratic 5% **B** 5 min, 5-95% **B** over 10 min, 95% **B** 5 min
M04	13.05	Isocratic 5% **B** 5 min, 5-95% **B** over 10 min, 95% **B** 5 min

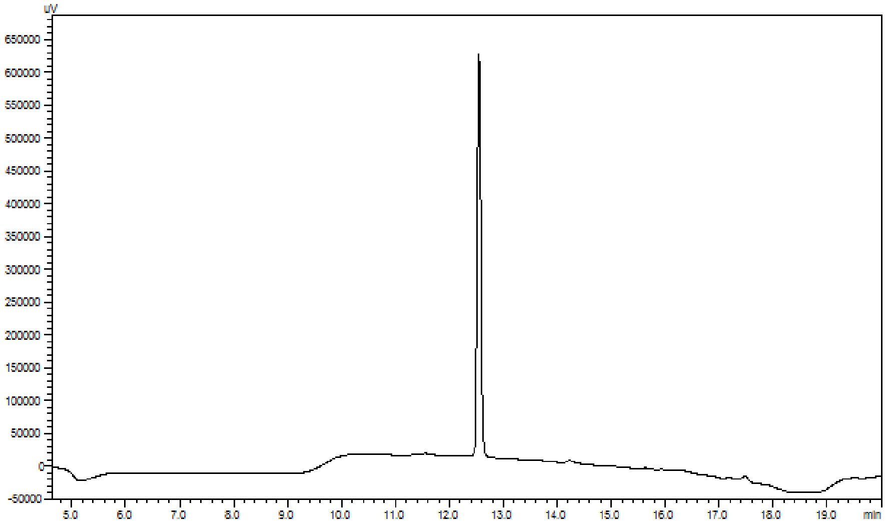

Figure S8. Analytical HPLC trace M02.

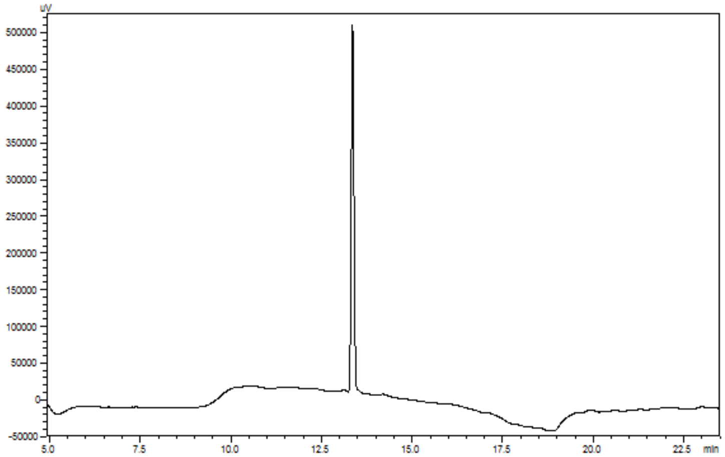

Figure S9. Analytical HPLC trace of M03.

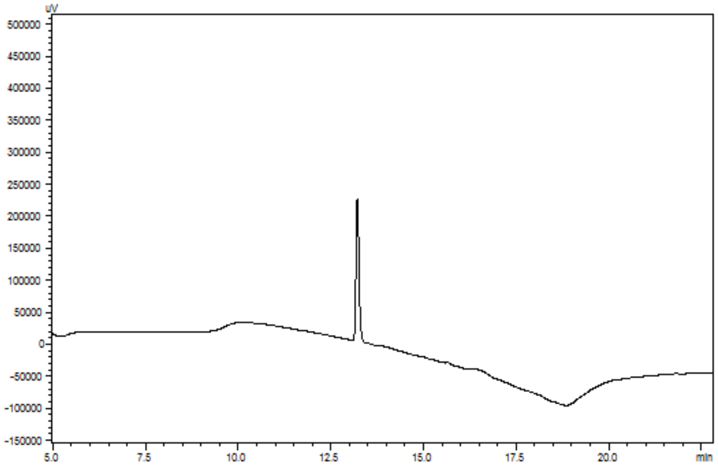

Figure S10. Analytical HPLC trace of M04.

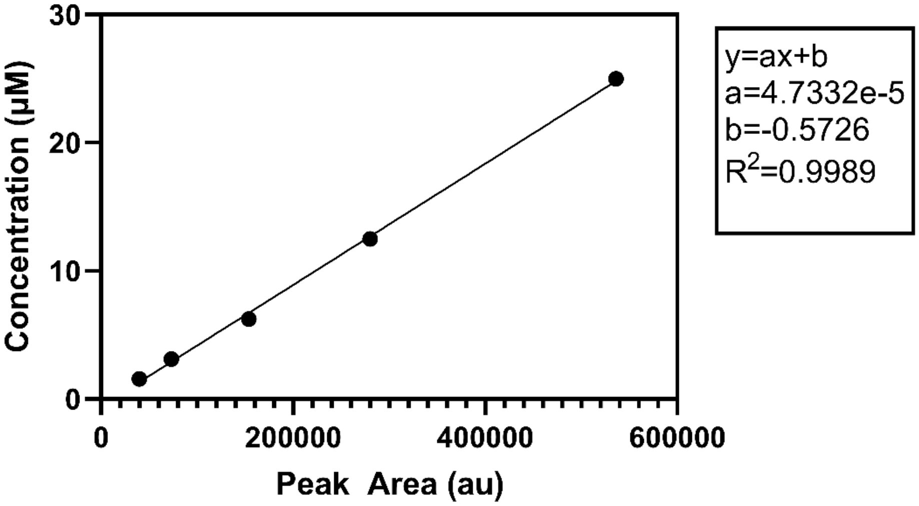

Figure S11. Representative analytical HPLC concentration curve of M02.

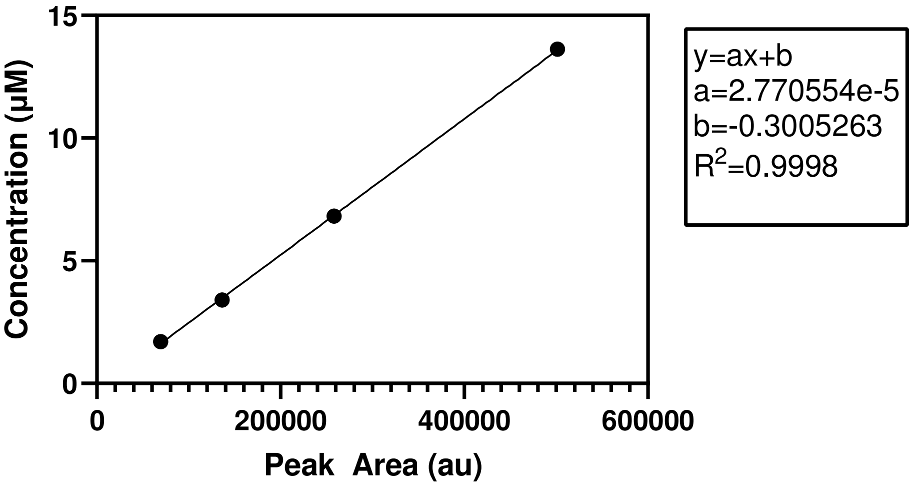

Figure S12. Representative analytical HPLC concentration curve for M03 and M04.

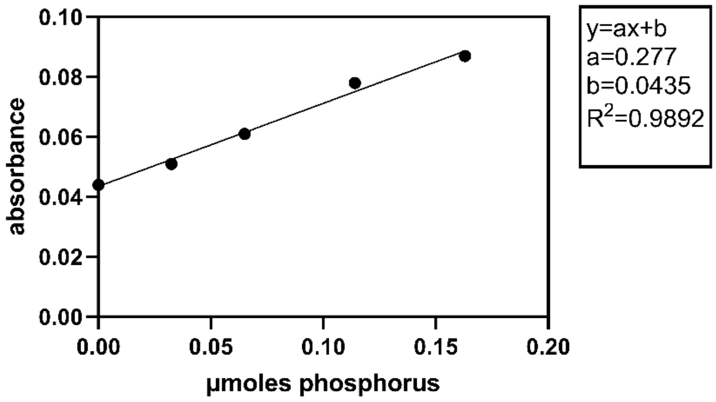

Figure S13. Representative phosphorus concentration curve.
